# Increased migratory activity and cartilage regeneration by superficial-zone chondrocytes in enzymatically treated cartilage explants

**DOI:** 10.1186/s12891-022-05210-2

**Published:** 2022-03-16

**Authors:** Yuichiro Shiromoto, Yasuo Niki, Toshiyuki Kikuchi, Yasuo Yoshihara, Takemi Oguma, Koichi Nemoto, Kazuhiro Chiba, Arihiko Kanaji, Morio Matsumoto, Masaya Nakamura

**Affiliations:** 1 Department of Orthopedic Surgery, School of Medicine, Keio University School of Medicine, 35 Shinano-machi, Shinjuku-ku, Tokyo, 160-8582 Japan; 2 Department of Orthopedic Surgery, National Defense Medical College, 3-2 Namiki, Tokorozawa City, Saitama, 359-8513 Japan; 3 Department of Orthopedic Surgery, National Hospital Organization, Murayama Medical Center, 2-37-1 Gakuen, Musashimurayama City, Tokyo, 208-0011 Japan

**Keywords:** Cartilage regeneration, Migratory activity, Superficial-zone chondrocyte, Lubricin, Platelet-derived growth factor

## Abstract

**Background:**

Limited chondrocyte migration and impaired cartilage-to-cartilage healing is a barrier in cartilage regenerative therapy. Collagenase treatment and delivery of a chemotactic agent may play a positive role in chondrocyte repopulation at the site of cartilage damage. This study evaluated chondrocyte migratory activity after enzymatic treatment in cultured cartilage explant. Differential effects of platelet-derived growth factor (PDGF) dimeric isoforms on the migratory activity were investigated to define major chemotactic factors for cartilage.

**Methods:**

Full-thickness cartilage (4-mm^3^ blocks) were harvested from porcine femoral condyles and subjected to explant culture. After 15 min or 60 min of actinase and collagenase treatments, chondrocyte migration and infiltration into a 0.5-mm cartilage gap was investigated. Cell morphology and lubricin, keratan sulfate, and chondroitin 4 sulfate expression in superficial- and deep-zone chondrocytes were assessed. The chemotactic activities of PDGF-AA, −AB, and -BB were measured in each zone of chondrocytes, using a modified Boyden chamber assay. The protein and mRNA expression and histological localization of PDGF-β were analyzed by western blot analysis, real-time reverse transcription polymerase chain reaction (RT-PCR), and immunohistochemistry, and results in each cartilage zone were compared.

**Results:**

Superficial-zone chondrocytes had higher migratory activity than deep-zone chondrocytes and actively bridged the cartilage gap, while metachromatic staining by toluidine blue and immunoreactivities of keratan sulfate and chondroitin 4 sulfate were detected around the cells migrating from the superficial zone. These superficial-zone cells with weak immunoreactivity for lubricin tended to enter the cartilage gap and possessed higher migratory activity, while the deep-zone chondrocytes remained in the lacuna and exhibited less migratory activity. Among PDGF isoforms, PDGF-AB maximized the degree of chemotactic activity of superficial zone chondrocytes. Increased expression of PDGF receptor-β was associated with higher migratory activity of the superficial-zone chondrocytes.

**Conclusions:**

In enzymatically treated cartilage explant culture, chondrocyte migration and infiltration into the cartilage gap was higher in the superficial zone than in the deep zone. Preferential expression of PDGF receptor-β combined with the PDGF-AB dimeric isoform may explain the increased migratory activity of the superficial-zone chondrocytes. Cells migrating from superficial zone may contribute to cartilage regeneration.

**Supplementary Information:**

The online version contains supplementary material available at 10.1186/s12891-022-05210-2.

## Background

Chondrocytes have been considered to be the only cell type present in articular cartilage; however, the zonal environment affects the morphology, proliferative, and migratory activity of chondrocytes. The superficial zone of articular cartilage contains disc-shaped chondrocytes oriented with their long axis parallel to the surface; the middle transitional zone includes oval chondrocytes dispersed singly or assembled in small groups; and the deep radial zone includes chondrocytes arranged in columns perpendicular to the articular surface [1–3]. Notably, both migratory activity and cartilage regenerative capacity are characteristic features of superficial-zone chondrocytes [4]. Recently, chondrocytes in the superficial zone have been reported to contain a population of cells with a progenitor-like phenotype [5–8]. These cells are characterized by having stem-cell-related surface markers, self-renewal ability, and the ability to differentiate into multiple lineages. One of the most notable markers is lubricin, the product of the *PRG4* gene [9–11]. The production and accumulation of lubricin is evident in cells of the superficial zone of articular cartilage as well as in other surface-lining cells of the meniscus and synovium [9, 11, 12].

Normally, chondrocytes do not migrate readily, given the surrounding dense, proteoglycan-rich pericellular matrix. In the context of cartilage injury, subsequent proteoglycan degradation may trigger chondrocyte migration from intact sites to remote sites of injury [13]. In clinical settings, transplantation of osteochondral cylinders to the cartilage defect sometimes fails to provide lateral integration of the graft cartilage with the adjacent host cartilage, creating a risk of further progressive cartilage degeneration over time [14]. Collagenase treatment of poorly integrated boundary sites between the graft and host cartilage has been shown to facilitate chondrocyte migration and cartilage fusion [15]. In full-thickness cartilage explant culture, short-term enzymatic digestion of the matrix can induce chondrocyte migration into the injured site, mimicking the physiological process of cartilage repair [16].

Platelet-derived growth factor (PDGF) is a potent mitogenic and chemotactic factor for cells of mesenchymal origin, including chondrocytes and mesenchymal stem cells. The four PDGF chains (denoted A to D) can assemble into five different dimeric isoforms, designated PDGF-AA, PDGF-BB, PDGF-AB, PDGF-CC, and PDGF-DD. Among these isoforms, PDGF-AB, −BB, and -CC can bind to and activate both PDGF receptor-α and -β (PDGFR-A and -B, respectively) [17–20], and PDGF-AB and -BB play a crucial role as chemotactic factors for chondrocytes [18]. Upon binding of PDGF ligands, PDGF receptors transduce signals to regulate multiple biological functions, including migration [21, 22].

The present study sought to evaluate chondrocyte behavior after enzymatic treatment of cartilage explants, and to compare chondrocyte migratory activity between cells derived from the superficial and deep zones. In addition, since PDGF is one of the most potent chemoattractants for inducing chondrocytes to populate the regions of damaged cartilage, the differential effects of various dimeric isoforms of PDGF on the migration of chondrocytes into the superficial and deep zones of cartilage were investigated.

## Methods

### Chondrocyte migration in enzymatically treated cartilage explant

**Fig. 1:**
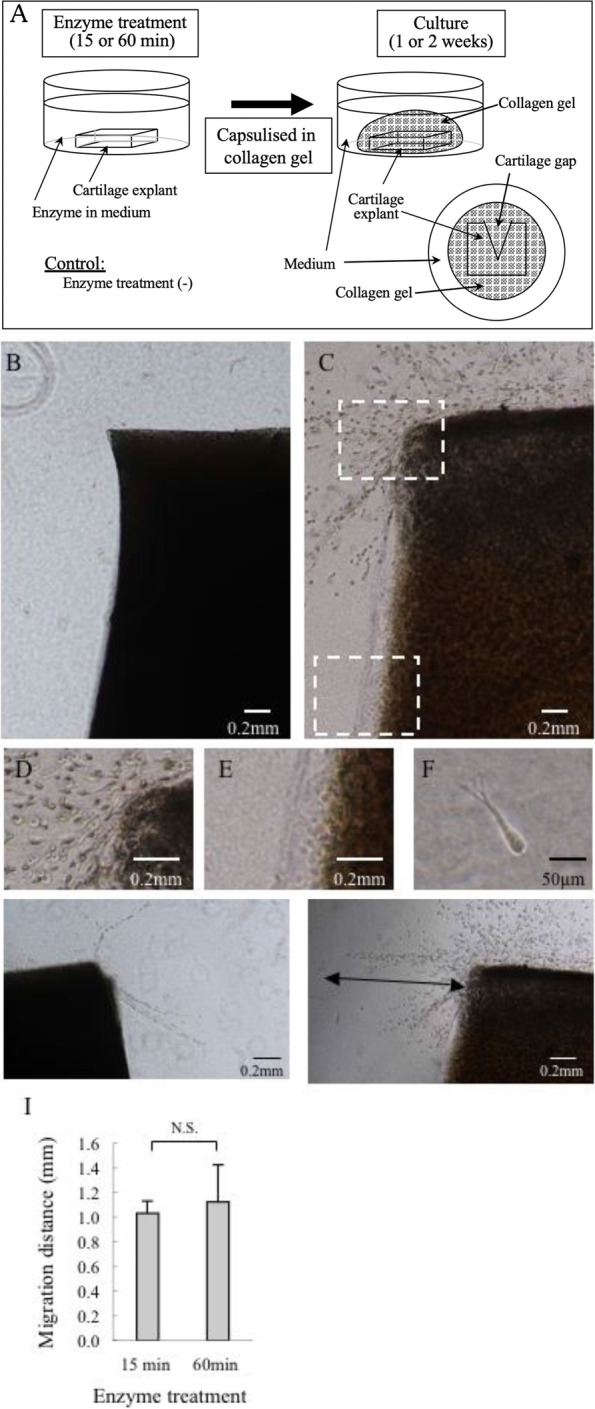
Culture of full-thickness cartilage explants and analysis of the effects of enzymatic treatment on chondrocyte migration. Cartilage explants harvested from the patellar articular cartilage of a 3-month-old pig were enzymatically treated (0.2% actinase and 0.02% collagenase P) in culture medium (Dulbecco’s modified essential medium supplemented with 10% fetal bovine serum, 0.2% ascorbic acid, and 0.5% antibiotics) for 15 or 60 min, encapsulated in type-I collagen gel, and cultured for 1 or 2 weeks (**A**). Cell migration was examined using a phase contrast microscope. No migrating chondrocytes were found without enzymatic treatment (**B**). Abundant cells migrated from the superficial zone of a cartilage explant treated with actinase and collagenase (**C**). Hyper magnification of the inset in **C** showing abundant cells migrating from the superficial zone (**D**) and no cells migrating from the deep zone (**E**). Hyper magnification of a representative migrating cell showing spindle shape with pseudopodia (**F**). Migration distance and the number of migrating cells were compared between the 15-min treatment group (**G**) and the 60-min treatment group (**H**). Arrow indicates migratory distance. Migratory distance was compared between the two groups (**I**). N.S.: not significant (The Mann-Whitney U test)

Full-thickness cartilage was harvested from the patella of a 3-month-old (young adult) pig; cartilage was obtained as rectangular blocks, each measuring 2 mm × 2 mm × 1 mm in size, that were generated by manually sawing. A 0.5-mm-diameter cartilage gap was created in each cartilage block. The cartilage blocks then were treated enzymatically with 0.2% actinase (Catalog 650,164, Kaken, Tokyo, Japan) and 0.02% collagenase P (Catalog 11,213,865,001, Sigma-Aldrich, St. Louis, MO, USA) in Dulbecco’s modified essential medium (DMEM; Sigma-Aldrich D5796) supplemented with 10% fetal bovine serum (FBS; Sigma-Aldrich), 0.2% ascorbic acid, and 0.5% antibiotics (streptomycin 1.0 mg/mL, penicillin 10,000 unit/mL), at 37 °C in a 5% CO_2_ atmosphere for 15 or 60 min. Blocks without enzyme treatment served as controls. The blocks with or without enzyme treatment were washed three times in serum-free DMEM. To permit visualization of the number and the migration distance of the migrating chondrocytes, the washed blocks were encapsulated in collagen gel using a collagen gel culturing kit (Nitta Gelatin, Osaka, Japan) and then incubated at 37 °C in a 5% CO_2_ atmosphere for 1 week (Fig. 1A). The blocks then were fixed with 4% paraformaldehyde and observed using a phase-contrast microscope (ECLIPSE TS 100, Nikon, Tokyo, Japan). Chondrocyte migration was examined at the outer cut edge or within the gap of the cartilage blocks. Next, the samples were embedded in a tissue-freezing medium and stained with toluidine blue. Some samples also were subjected to immunohistochemical analysis (see below).

### Migration of isolated chondrocytes using Boyden chambers

Chondrocytes were isolated from the knee of a 3-month-old pig. Superficial-zone (shallowest one-third) and deep-zone (deepest one-third) cartilage samples were dissected away from the femoral condyle. Cartilage samples were digested with actinase as above but for 2 h with stirring, followed by digestion (overnight at 37 °C in a 5% CO_2_ atmosphere) with collagenase (Sigma-Aldrich) at a final concentration of 0.02% in serum-free DMEM (1 mL per well). The density of the chondrocytes was determined by counting with a hemocytometer and set to 10^5^ cells per mL. Migration assays were performed as follows. A modified Boyden chamber (Becton, Dickinson and Company, Franklin Lakes, NJ, USA) was prepared using a 12-well cell culture insert companion plate and cell culture insert containing polyethylene terephthalate membrane with 8-μm pores. The lower chambers were filled with serum-free DMEM, and the upper chambers (inserts) were placed over the lower chambers. Chondrocytes in serum-free DMEM were placed into the upper inserts and the plates were incubated overnight at 37 °C in a 5% CO_2_ atmosphere to permit the cells to attach to the membrane. Next, the upper inserts were transferred to fresh lower chambers containing serum-free DMEM (control) or DMEM supplemented with 10% FBS, PDGF-AA (50 ng/mL), PDGF-AB (50 ng/mL), or PDGF-BB (50 ng/mL). Cells were allowed to migrate toward the lower side of the insert membrane during a 6-h incubation at 37 °C in a 5% CO_2_ atmosphere. After incubation, cells remaining on the upper surface (having not migrated through the membrane) were removed from the upper surface by wiping with a cotton swab, and membranes then were removed, fixed with 4% paraformaldehyde, stained with Meyer’s hematoxylin, and mounted on glass slides. The number of migrated cells was counted in three randomly selected fields per membrane using a light microscope at 20× magnification, and the mean and standard deviation (SD) were calculated for each treatment.

### Gene expression

Total RNA was extracted from various portions of cartilage using a RNeasy Lipid Tissue Mini Kit (Qiagen, Valencia, USA) according to the manufacturer’s instructions. An aliquot of each resulting total RNA preparation (in a reaction volume of 20 μL) was reverse transcribed into cDNA using a High Capacity cDNA Reverse Transcription kit (Applied Biosystems [ABI], Foster City, CA, USA). The resulting single-stranded cDNA products then were analyzed by real-time (RT) PCR using TaqMan gene expression assays (ABI) and an ABI 7900HT Fast Real Time PCR System (ABI). Specifically, cDNA samples (1 μL in a 21-μL reaction volume) were analyzed for the genes of interest. The master-mix consisted of 1 μL of 20x TaqMan® Gene Expression Assays (ABI), 9 μL of water, 10 μL of 2x TaqMan® Gene Expression Master Mix (ABI), and 1 μL (18 μM) of PCR primers (Invitrogen, Waltham, MA, USA). The level of expression of each target gene was normalized to that of *GAPDH* (encoding glyceraldehyde dehydrogenase, a housekeeping protein) in the same sample, and expression then was compared between samples. Analysis of each sample was repeated 5 times for each gene of interest. RT-PCR was performed using a program as follows: samples were initially denatured at 95 °C for 20 s, then subjected to 40 cycles of denaturation at 95 °C for 1 s, annealing at the primer-specific temperature for 30 s, and elongation at 60 °C for 20 s. The gene of interest was *PDGFRB*, which encodes PDGFR-B. The sequence of the pig *PDGFRB* gene transcript was retrieved from GenBank as Accession No. AK394805.1, and primers and probes for the target were designed using Primer3 (http://primer3.sourceforge.net/). To avoid genomic DNA amplification in the PCR results, the exon-intron boundaries were confirmed by comparison of the RNA sequence with the genomic sequence from Blat at UCSC (http://genome.ucsc.edu). The probe and primer sequences for *PDGFRB* were as follows: probe, 5′-AAT GAA GTC AAC ACC GCC TC-3′; forward primer, 5′-CAA CGA GGG TGA CAA CGA CT-3′; and reverse primer, 5′-CTC TGG CTC TGG CTC CTC TT-3′. The difference in threshold cycle (ΔCt) for target sequences versus *GAPDH* (ABI Ss03375435)101 was measured for each sample.

### Western blot analysis

Approximately 200 mg wet weight of various portions of cartilage were incubated for 24 h at 4 °C on a rocker after addition of 600 μL of cold buffer (50 mM Tris-HCl, pH 8.0, 150 mM NaCl, 0.5% (w/v), NP40, 0.5% (w/v) deoxycholic acid, and 1 tab of proteinase inhibitor (Complete Mini, Catalog 11,836,153,001, Roche, Basel, Switzerland). The samples were cleared by centrifugation at 10,000–15,000 rpm at 4 °C for 20 min, and each cleared supernatant was combined with 3 volumes of cold absolute ethanol and incubated overnight at − 20 °C. The samples then were centrifuged at 10,000–15,000 rpm at 4 °C for 20 min, and each resulting pellet was washed, dried, and resuspended in 1 mL of chondroitinase ABC (CHase) buffer (50 mM sodium acetate, 50 mM Tris, 10 mM EDTA, pH 7.6). Each extract then was combined with 15 μL (60 mU) of CHase and the mixtures were incubated for 2 h at 37 °C to dissolve the samples. For western blotting, protein extract samples were separated on a 10% SDS-PAGE gel and electroblotted onto nitrocellulose membranes. After blocking with 1% Membrane Blocking Agent (Catalog RPN2125V; GE Healthcare, Boston, MA, USA) membranes were incubated overnight at 4 °C with 1000-fold diluted rabbit monoclonal anti-PDGFR-B antibody (Catalog #4564, Clone No. C82A3; Cell Signaling Technology, Danvers, MA, USA) or 2000-fold diluted anti-tubulin antibody (Catalog #9280-0050G, BIO-RAD, Hercules, CA, USA). After washing, membranes were incubated for another hour with the secondary antibody (1000-fold diluted rabbit monoclonal anti-PDGFR-B antibody or 2000-fold diluted anti-tubulin antibody). Membranes were washed and rinsed with ECL™ Prime Western Blotting Detection Reagent (Catalog RPN2236; GE Healthcare). Bands were imaged using LAS-3000 (Fujifilm, Tokyo, Japan), followed by quantification of band intensity using Image J (ver. 5.0, National Institute of Health, USA).

### Immunohistochemistry

Staining was carried out using anti-lubricin antibody (Catalog 5C11; Sigma-Aldrich), anti-PDGFR-B antibody (as above), anti-keratan sulfate antibody (Catalog RIT-M001, Cosmo Bio Co., Ltd., Tokyo, Japan), and anti-chondroitin 4 sulfate antibody (Catalog PRPG-BC-M02, Cosmo Bio Co., Ltd.) for immunostaining. Cartilage explants (for staining with anti-lubricin antibody, anti-keratan sulfate antibody, and anti-chondroitin 4 sulfate antibody) were obtained from the medial condyle of a 3-month-old pig. Similarly, whole osteochondral tissues (for staining with anti-PDGFR-B antibody) were harvested from the medial condyle of the tibia of a 3-month-old pig and consisted of tissue from the peripheral region that was covered with meniscus to the central region that was not covered with meniscus and close to the intercondylar eminence of the tibia. Cartilage explants were treated enzymatically and cultured for 1 or 2 weeks as described above. These explants then were fixed in 10% neutral buffered formalin for 3 days and embedded in paraffin wax. Before embedding, osteochondral tissues were decalcified by incubation for 14 days in 5% ethylenediaminetetraacetic acid (EDTA) in phosphate-buffered saline (PBS). Sections of the resulting samples were deparaffinized in xylene and dehydrated in ethanol. Endogenous peroxidase activity was blocked by incubation in 0.6% H_2_O_2_. Peroxidase-labeled polymer (Catalog K4003; DAKO, Santa Clara, CA, USA) and diaminobenzidine (DAB) substrate solutions were used for visualization of immunoreactivity. Samples were observed using a light microscope (AXIO Imager A1, Carl Zeiss Microscopy, Jena, Germany).

### Statistical analysis

The Mann-Whitney U test was used for comparisons between two groups. Multiple comparisons were performed by one-way analysis of variance (ANOVA) followed by a Bonferroni post hoc test.**:***p* < 0.05 was considered statistically significant. All statistical analyses were performed using SPSS software (version 17.0; SPSS, Chicago, USA).

### Ethics statement

All procedures were conducted in accordance with Institutional Guidelines on Animal Experimentation at Keio University and Institutional Guidelines on Animal Experimentation at the National Defense Medical College. Experimental approval was obtained from an institutional review board of Keio University and the National Defense Medical College. This study was carried out in compliance with the ARRIVE guidelines.

## Results

### Migration of chondrocytes from enzymatically treated cartilage explants

After collagenase and actinase treatment for 60 min followed by 1-week culture, chondrocytes were observed to migrate from the outer edge of full-thickness cartilage explants into the collagen gel (Fig. 1C), whereas chondrocytes did not migrate from non-treated cartilage explants (Fig. 1B). Chondrocytes migrated from the superficial zone, but not from the deep zone despite identical treatment with collagenase and actinase (Fig. 1D, E). Morphologically, the chondrocytes migrating into the collagen gel had an elongated appearance with pseudopodia (Fig. 1F). When focusing on the effects of enzyme treatment period on the cell number and the distance migrated from the cartilage outer edge, the 60-min enzyme treatment yielded an apparently larger number of migrated chondrocytes than seen with the 15-min treatment, but the distance migrated was comparable between the two treatment periods (Fig. 1G-I).

**Fig. 2:**
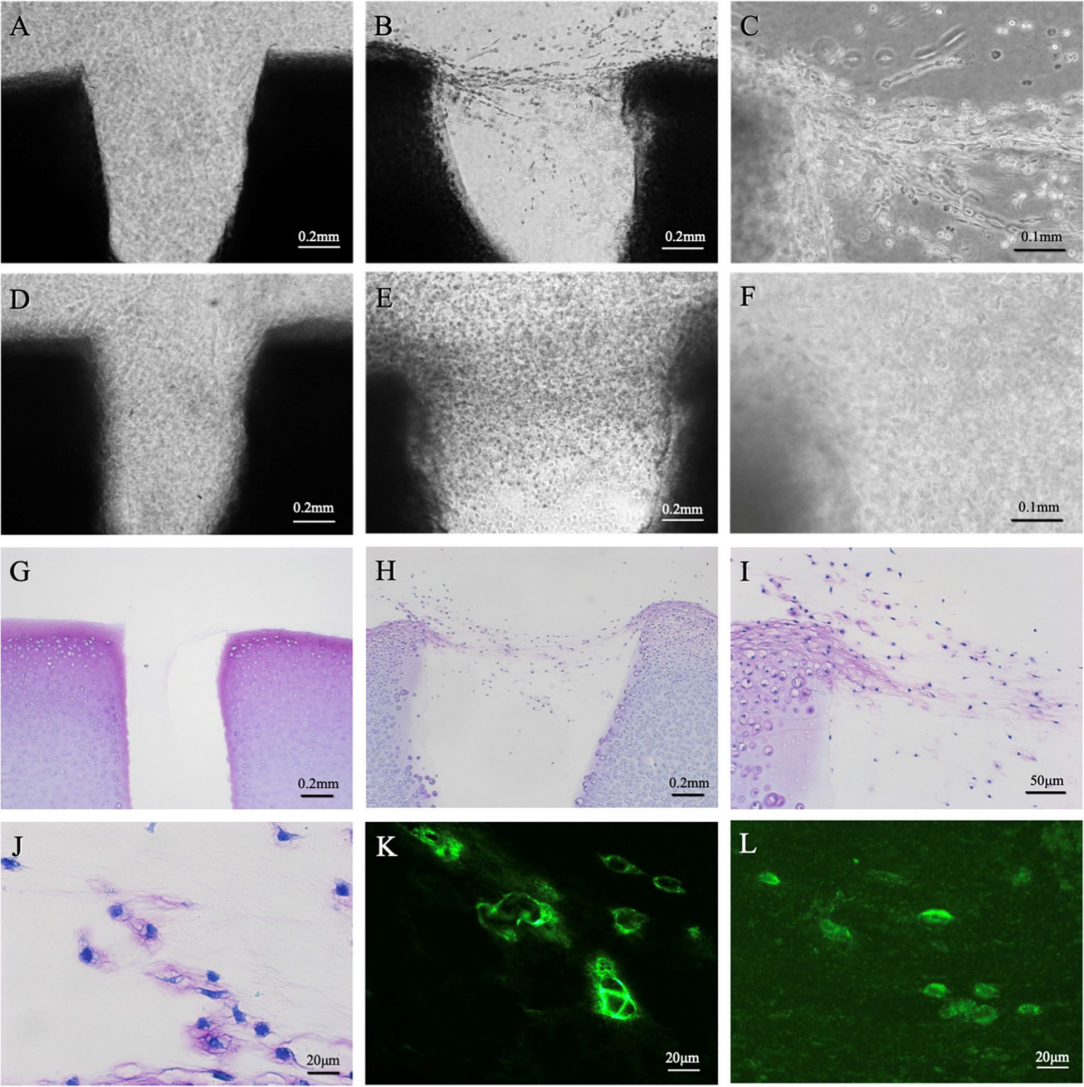
A cartilage gap was created in the full-thickness cartilage explant, and the effects of enzymatic treatment on chondrocyte migration were analyzed. Under the phase contrast microscope, within the gap, no chondrocytes migrated out of cartilage explant in the absence of enzyme treatment, as assessed after culturing for 1 week (A: × 4) or 2 weeks (D: × 4). After 60 min of enzyme treatment followed by 1 week (B: × 4, C: × 10) or 2 weeks (E: × 4, F: × 10) of culturing, the cartilage gap was bridged and filled with abundant chondrocytes migrating from the superficial zone. After 60 min of enzyme treatment followed by 2 weeks of culturing, metachromasia for toluidine blue(H: × 4, I: × 10, J: × 40) and immunoreactivities for keratan sulfate (K: × 40) and chondroitin 4 sulfate (L: × 40) were found around migrating cells. Cartilage explant in the absence of enzyme treatment served as a control (G: × 4)

When a 0.5-mm-diameter gap was created in the full-thickness cartilage explant, followed by 60 min of enzymatic treatment and 1 week (Fig. 2B, C) or 2 weeks (Fig. 2E, F) of culturing, chondrocytes migrated out of the superficial zone and bridged the cartilage gap. In contrast, no cells migrated when the experiment was performed using control cartilage explants that had not been enzymatically treated (Fig. 2A, D). After 60 min of enzyme treatment followed by 2 weeks of culturing, the migrating cells showed metachromatic staining with toluidine blue (Fig. 2H, I, and J), whereas no metachromatic staining was observed in the control (Fig. 2G). Positive immunoreactivities to keratan sulfate and chondroitin 4 sulfate were found around migrating cells (Fig. 2K, L).

**Fig. 3:**
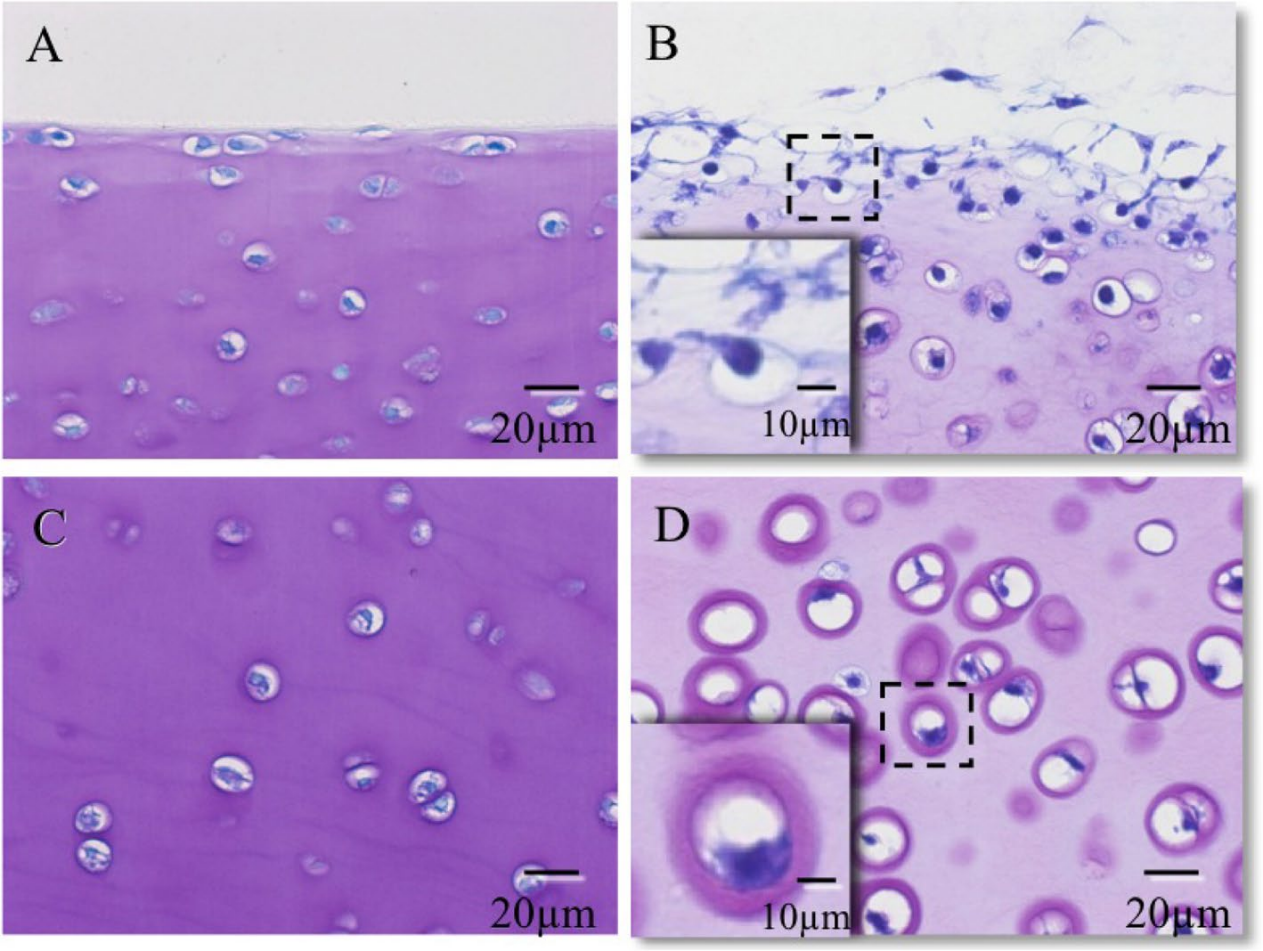
Toluidine blue staining of cultured cartilage explant without (**A**, **C**) and with 60-min enzyme treatment (**B**, **D**). Marked proteoglycan loss and subsequent chondrocyte migration from the lacuna in the superficial zone were observed (B). Metachromasia of the cartilage matrix was weakened, but cellular migration from the lacuna was not observed, in the deep zone (**D**). Hyper-magnification of the dashed box indicates that proteoglycan content appeared to be sufficient to prevent cell migration (**D**)

Toluidine blue staining of enzyme-treated cartilage explants revealed the loss of proteoglycan, especially in the superficial zone; the chondrocytes were observed to migrate out of these lacunae and to extend pseudopodia (Fig. 3B). In contrast, in the deep zone, the pericellular matrix surrounding the chondrocytes remained sufficient to prevent cell migration (Fig. 3D). Toluidine blue staining of cartilage explant without enzyme treatment revealed no loss of proteoglycan and the chondrocytes remained in the pericellular matrix in both zones (Fig. 3A, C).

**Fig. 4:**
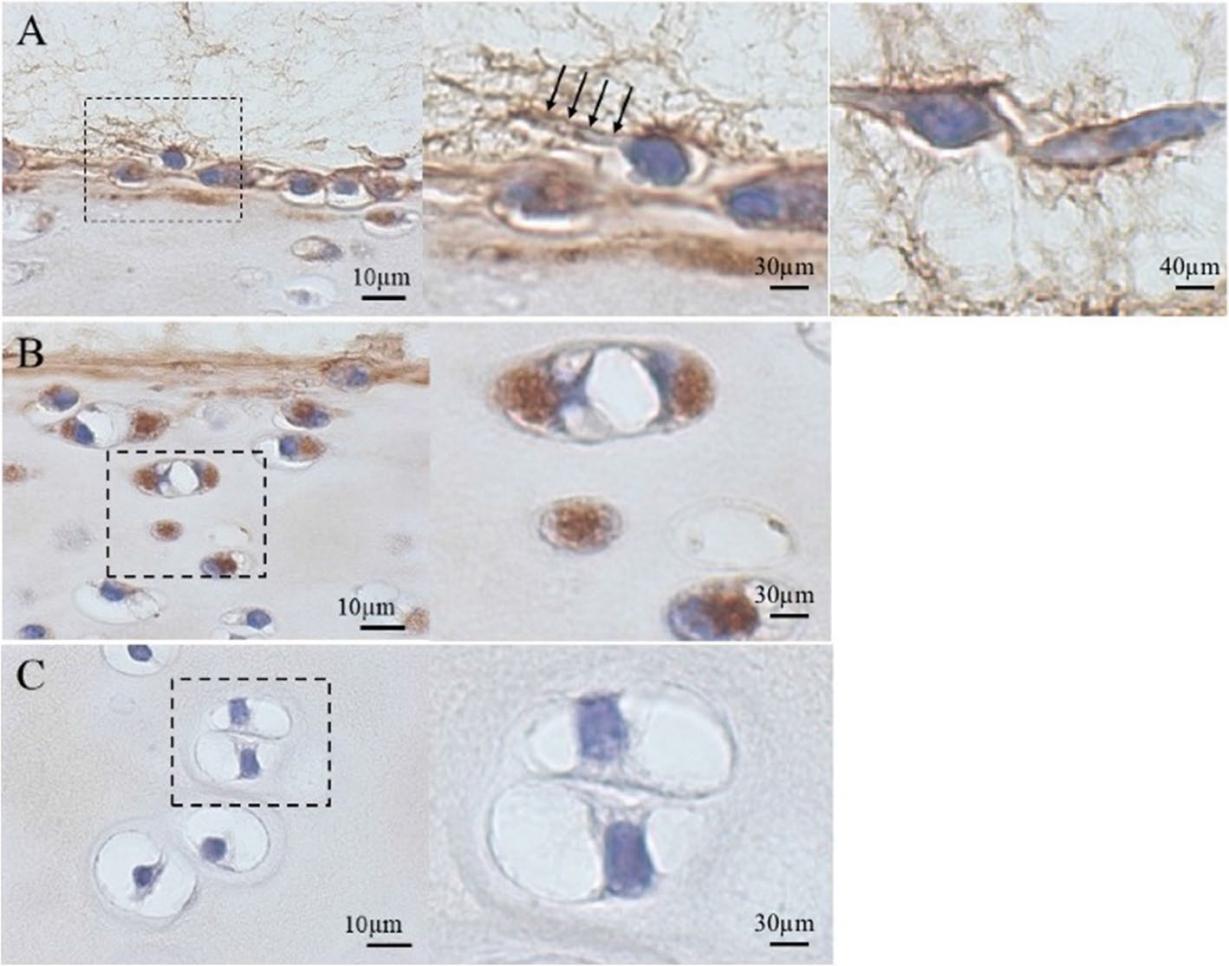
Immunolocalization of lubricin in enzyme-treated cartilage explant after 1-week culture. Immunoreactivity for lubricin was positive in the superficial- to middle-zone chondrocytes (**A**, **B**), but not in the deep-zone chondrocytes (**C**). Lubricin expression was not apparent in migrating chondrocytes with elongated shape (A, right panel). The inset of the left panel shows a cell preparing to migrate out from the superficial zone. Arrows indicate pseudopodia (A, middle panel). Right panels of B and C indicate hyper-magnification of the dashed boxes in the respective left panels

Immunohistochemical staining of enzyme-treated and 1-week-cultured cartilage explants demonstrated increased intracellular immunoreactivity for lubricin in chondrocytes of the superficial and middle zones (Fig. 4B). However, immunoreactivity for lubricin was not apparent in the elongated cells undergoing migration or those that appeared to be preparing to migrate out of the superficial zone (Fig. 4A, middle and right panels) nor in the deep-zone chondrocytes (Fig. 4C).

### Differential effects of PDGF dimeric isoforms on chondrocyte migration in Boyden chambers

**Fig. 5:**
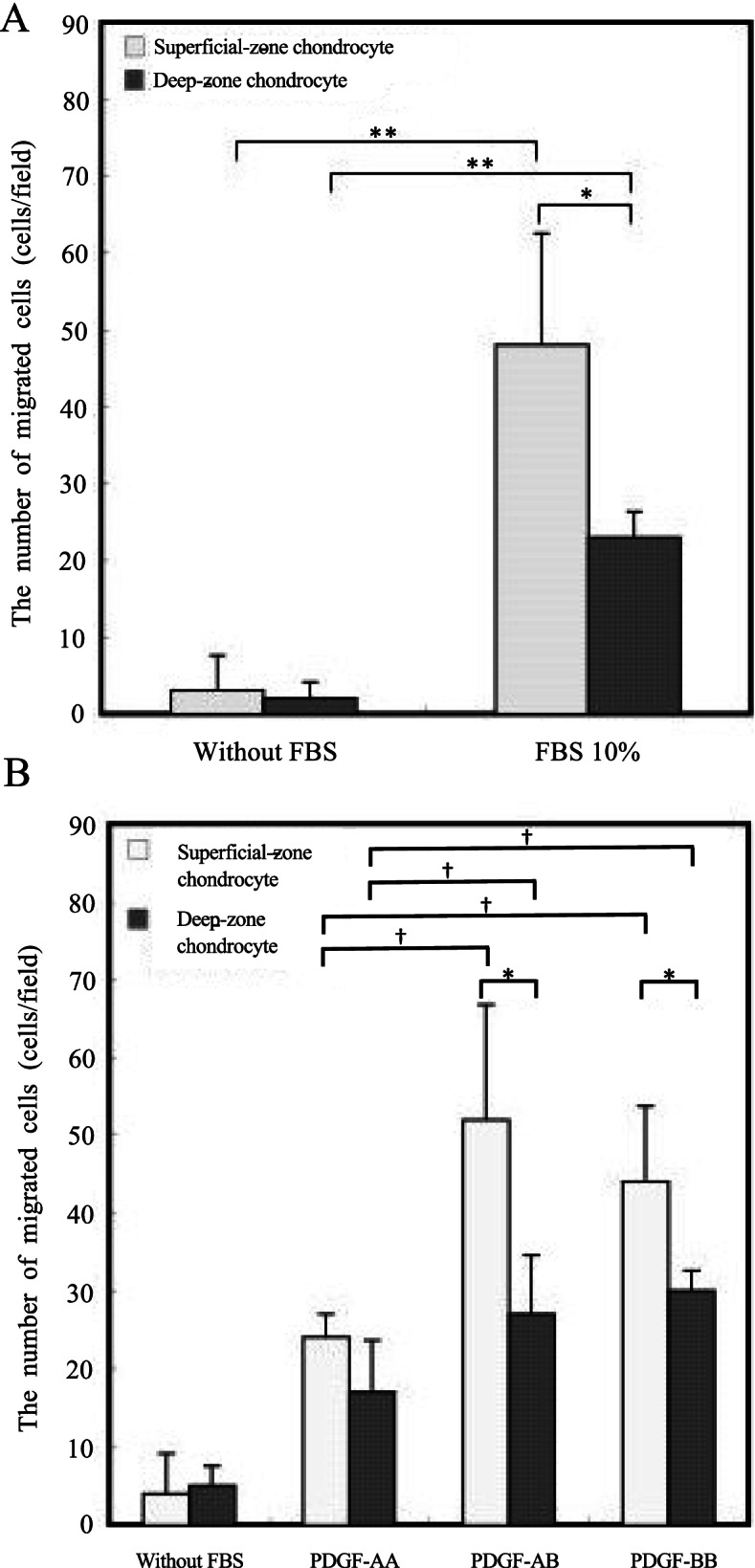
Migration of isolated chondrocytes assessed using a Boyden chamber. The number of migrated chondrocytes was significantly larger in the medium supplemented with FBS than in medium without FBS. (*p < 0.05, **p < 0.01; Mann-Whitney U-test) (A). Among PDGF dimers, the number of migrated cells was larger in the presence of PDGF-AB and -BB than in the presence of PDGF-AA (*p < 0.05; Mann-Whitney U-test, **† < 0.05, One-way ANOVA and Bonferroni post-hoc test.) (B)

The number of migrated chondrocytes was significantly larger for explants incubated in growth medium supplemented with 10% FBS than in medium lacking FBS. In addition, the number of chondrocytes migrating from the superficial zone was significantly larger than that of chondrocytes migrating from the deep zone (Fig. 5A). When the effects of PDGF isoforms were analyzed, the effects were small compared to those of zonal difference. Regardless of PDGF isoform, the number of migrated cells in the superficial zone was consistently higher than that of cells in the deep zone. Among PDGF dimers, PDGF-AB and -BB exerted larger effects than PDGF-AA (*p* < 0.05, Fig. 5B).

### Preferential PDGFR-B mRNA and protein expression in the superficial zone

**Fig. 6:**
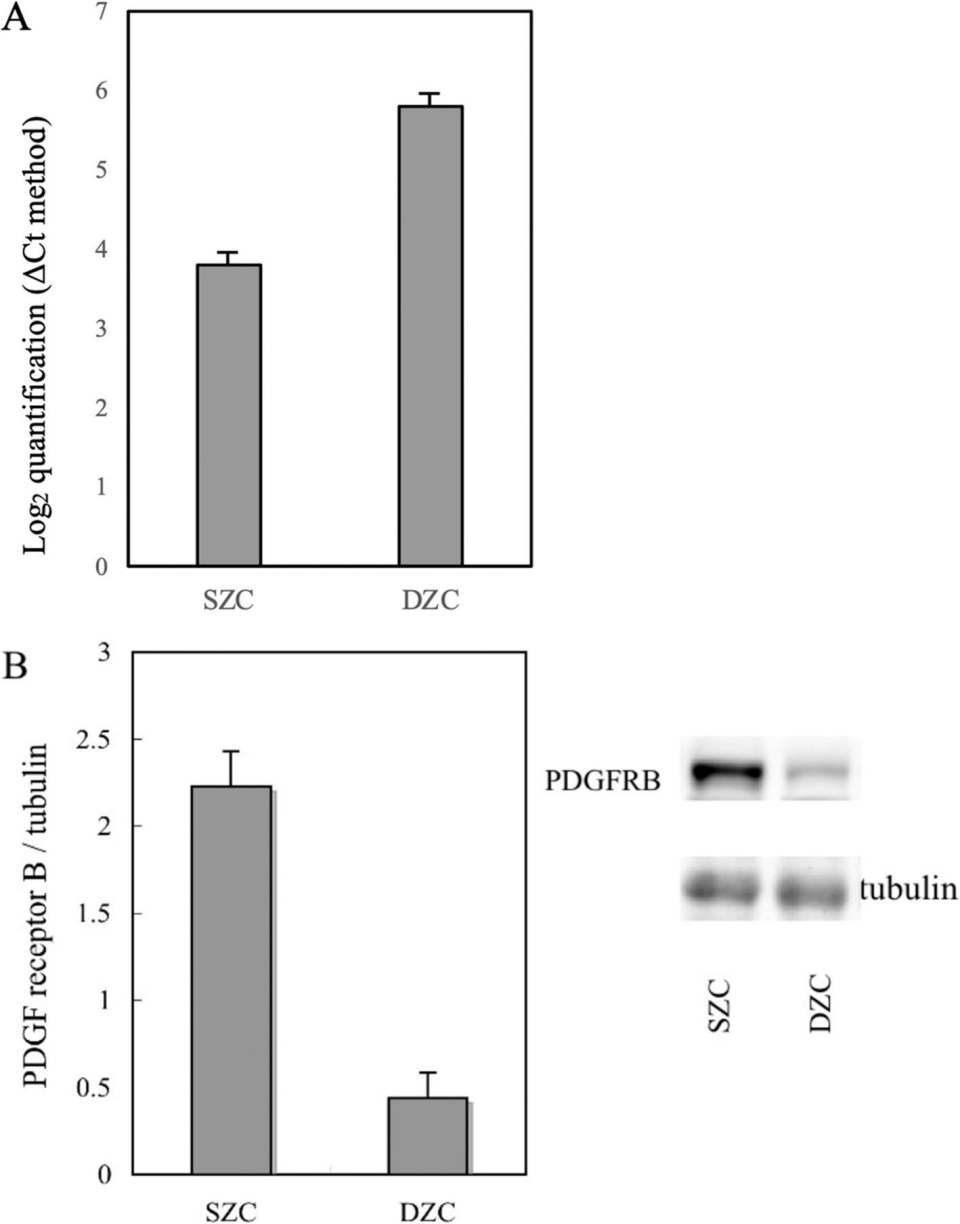
mRNA and protein levels of PDGF receptor-β produced by chondrocytes in superficial and deep zones. *PDGFRB* mRNA level in superficial-zone chondrocytes (SZC) was 4 times higher than that in deep-zone chondrocytes (DZC), as assessed by quantitative reverse transcription-PCR (ΔCt method) (**A**). The protein level of PDGF receptor-β was > 4 times higher in the superficial zone than in the deep zone, as assessed by Western blot analysis (**B**). The blots were cropped from different parts of the same gel; and full-length gels and blots are included in the Supplementary Information. **p < 0.01, Mann-Whitney U-test

*PDGFRB* mRNA levels were analyzed in chondrocytes from the superficial and deep zones. This transcript accumulated to 4-fold higher levels in superficial-zone chondrocytes than in deep-zone chondrocytes (Fig. 6A). Consistent with those results, western blotting analysis revealed that PDGFR-B, which was detected as a band of the expected size (180–190 kDa), accumulated to > 4-fold higher levels in superficial-zone chondrocytes than in deep-zone chondrocytes (Fig. 6B).

### PDGFR-B is highly expressed in the superficial-zone chondrocytes of the peripheral region

**Fig. 7:**
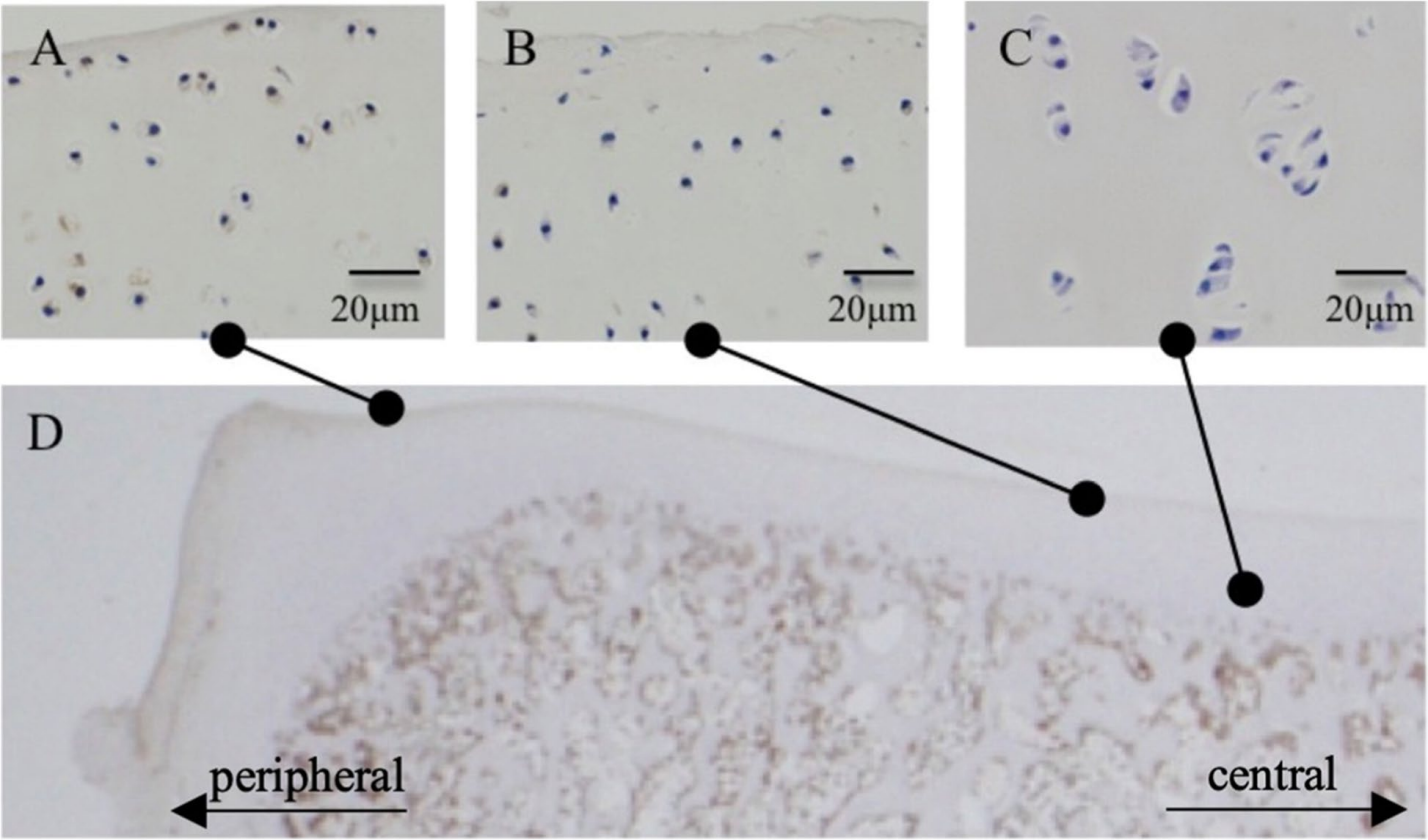
Histological localization of PDGF receptor-β. Osteochondral tissue was harvested from the medial condyle of the tibia of 3-month-old pig (**D**). Chondrocytes in the superficial zone showed positive staining for PDGF receptor-β in the peripheral region of the osteochondral tissue (**A**). In the central region of the osteochondral tissue, a few positive cells were observed in the superficial zone (**B**). No positive cells were detected in the deep zone (**C**)

Immunohistochemistry revealed that, in the peripheral region of osteochondral tissue (Fig. 7D) harvested from the medial condyle of tibia of a 3-month-old pig, many chondrocytes in the superficial zone showed positive staining for PDGFR-B (Fig. 7A). On the other hand, in the central region of the medial condyle, the number of positive cells was lower in the superficial zone, and positive cells were not seen in the deep zone (Fig. 7B, C).

## Discussion

In normal cartilage, chondrocytes are surrounded by a proteoglycan-rich, dense, pericellular matrix that renders these cells unable to migrate [13]. However, cells in mechanically damaged cartilage migrate to the injured site [4, 5], and cells in early-stage osteoarthritis (OA) express surface markers such as CD105 and CD166 [7] and exhibit stem-cell-related markers such as Notch-1, STRO-1, and vascular cell adhesion molecule-1 (VCAM-1) [1]. Therefore, it is possible that chondrocytes in injured cartilage or early-stage OA migrate out of the degraded collagen network, infiltrating the damaged site and contributing to cartilage repair.

In the present study, we successfully developed a chondrocyte migration model by sequential treatment of cartilage explants with actinase and collagenase followed by in vitro culture, consistent with the method reported by Seol et al. [16]. Our model has several advantages. First, the model mimics cartilage degeneration of the superficial layer. When the enzymatic treatment period was timed to control the degree of cartilage matrix degradation, migrating chondrocytes were recognized only in the superficial zone, where proteoglycan content is lower than that in the deep zone. Second, our model permitted the visualization not only of chondrocyte migration but also of chondrocyte infiltration into the cartilage gap. Embedding the cartilage explant within the collagen gel may facilitate visualization of the chondrocytes infiltrating the gap. In fact, Seol et al. [16] examined the effects of enzymatic treatment on chondrocyte behavior without embedding in collagen gel. In the present study, chondrocytes spontaneously moved to and fully bridged the cartilage gap, rather than randomly migrating in all directions. This observation may be attributable, at least in part, to cell-cell interaction, as reported by Honig et al. [23]. Moreover, the collagen gel appeared to act as a scaffold in the cartilage gap, stimulating cartilage regeneration [24]. Lastly, after enzymatic treatment, chondrocytes started to migrate from the superficial zone, and produced keratan sulfate and chondroitin 4 sulfate, potentially contributing to the repair of damaged cartilage. A few in vivo animal studies assessed the effects of collagenase treatment on cartilage repair. Gurer et al. tried to enhance cartilage regeneration in the knees of sheep by using collagen membrane soaked with collagenase [25]. Sueyoshi et al. revealed that collagenase treatment was effective on cubic micro-cartilage to expand cartilage regeneration capacity in ear cartilage tissue engineering [26]. With the described sequential enzyme treatment of collagenase and actinase in our study, it is possible that accelerated cartilage matrix synthesis and chondrocyte migration may promote beneficial effects in clinical settings.

Recently, chondrocytes in the superficial zone have attracted attention as a population believed to include cartilage stem/progenitor cells (CSPCs) that exhibit stem-cell-related markers [5–8, 27–29]. One of the most notable markers is lubricin, the product of the *PRG4* gene [9–11]. The production and accumulation of lubricin is evident in cells of the superficial zone of articular cartilage as well as in other surface-lining cells of the meniscus and synovium [9, 11, 12], but decreased expression of lubricin has been reported to correlate with OA progression [30, 31]. In the present study, lubricin production was apparent in chondrocytes preparing to migrate out from the superficial zone, but not in migrating elongated chondrocytes. In contrast to the case in chronic OA, the enzymatically treated cartilage explants of the present study retained active superficial-zone chondrocytes that possessed the ability to migrate into and repair damaged cartilage. As described earlier, our model mimicked cartilage degeneration, such that superficial-zone chondrocytes seemed to be intact, expressing lubricin abundantly and potentially participating in the repair of cartilage erosion; in contrast, deep-zone chondrocytes did not migrate due to the continued presence of a dense surrounding cartilage matrix.

Given that no cellular migration was observed in the deep zone, due to the difference of proteoglycan content between the superficial and the deep zones (presumably reflecting the interval of enzymatic treatment), we isolated (separately) chondrocytes from the superficial and deep zones for further characterization. Migratory activity of the two types of chondrocytes was assessed using a Boyden chamber. Our results demonstrated that PDGF-AB and –BB act as chemoattractants, such that stronger migratory activity in the presence of these factors was seen for the superficial-zone chondrocytes than for the deep-zone chondrocytes. This observation is consistent with literature reports indicating that, of the various PDGF dimers, PDGF-AB and -BB are the most potent chemoattractants for chondrocytes [2, 18, 32–34]. Additionally, it has been reported that there are two PDGF receptors, PDGFR-A and -B; the latter is believed to have a greater role in chondrocyte migration than the former [18]. In the present study, addition of exogenous PDGF-AB and -BB preferentially stimulated migratory activity of the superficial-zone chondrocytes (compared to deep-zone chondrocytes), and we showed that PDGFR-B mRNA and protein accumulate to higher levels in superficial-zone cells than in deep-zone cells. Therefore, the superior affinity of PDGF ligands for PDGFR-B may explain the increased migratory activity of superficial-zone chondrocytes in our model. These results are consistent with the observation that platelet-rich plasma, which contains high levels of PDGF, is a potential stimulator of cartilage regeneration in the clinical setting [35].

Finally, there are several limitations in this study. Firstly, it is difficult to conclude why superficial-zone chondrocytes exhibited migratory activity superior to that of deep zone chondrocytes in our explant culture model. The superficial cartilage matrix has lower proteoglycan content, so shorter enzymatic treatment may be better at effectively degrading and exposing superficial-zone chondrocytes compared to deep-zone chondrocytes. Additionally, we showed that PDGFR-B is expressed at higher levels in the superficial-zone chondrocytes. The number of lubricin-expressing cells with stem cell-like character was larger in the superficial zone. Collectively, multiple factors might be involved, preventing us from attributing the difference to a single reason. Secondly, our cartilage degeneration model was triggered by enzymatic treatment, rather than by mechanical stress. However, in human OA, persistent mechanical overload plays a critical role in the pathogenesis. Thirdly, in our experiments, each cartilage explant was encapsulated with collagen gel. Ideally, only the cartilage gap would be filled with collagen gel or scaffold; however, the filling of this space alone was difficult, given that the cartilage slice that was only 2 mm × 2 mm × 1 mm in size and that the gap was 0.5 mm in diameter. We currently are experimenting with ways of modifying the model to address this issue. Fourth, PDGF-AB and BB are reportedly more potent chemoattractants for articular chondrocytes [18], but PDGF-AA also acts, at least in part, as a chemoattractant for mesenchymal cells [31, 36, 37]. Therefore, the effects of the PDGF-AA/PDGFR-A interaction will need to be assessed in the various classes of chondrocytes. Lastly, in all of the experiments described here, cartilage tissue was harvested from 3-month-old pig just after euthanasia, so tissue viability was expected to be certain. However, tissue viability typically will need to be confirmed before subsequent experiments.

In conclusion, in our enzymatically treated cartilage degeneration model, superior chondrocyte migration and infiltration into the damaged region was observed from the superficial zone compared to that from the deep zone [2]. Migrated chondrocytes potentially contribute to matrix regeneration after cartilage degeneration. Increased PDGFRB expression combined with selective PDGF dimeric isoforms may explain, in part, the higher migratory activity of superficial-zone chondrocytes.

## Supplementary Information

**Additional file 1.**

## Data Availability

The datasets generated and/or analyzed during the current study are available in the GenBank repository (Accession Number: AK394805.1).
