# Supplementary material for: Increased migratory activity and cartilage regeneration by superficial-zone chondrocytes in enzymatically treated cartilage explants
Source: BMC Musculoskelet Disord. 2022 Mar 16;23:256. doi: 10.1186/s12891-022-05210-2 (PMC8925221; doi:10.1186/s12891-022-05210-2)
Supplement: Supplementary file 1 — Additional file 1. [file 12891_2022_5210_MOESM1_ESM.pdf]

Figure 6 Supplementary Information

Title

Increased migratory activity and cartilage regeneration by superficial zone chondrocytes in enzymatically treated cartilage explants

Authors list

Yuichiro Shiromoto[1 2], Yasuo Niki[1], Toshiyuki Kikuchi[3], Yasuo Yoshihara[2 3], Takemi Oguma[2], Koichi Nemoto[2], Kazuhiro Chiba[2], Arihiko Kanaji[1], Morio Matsumoto[1], Masaya Nakamura[1]

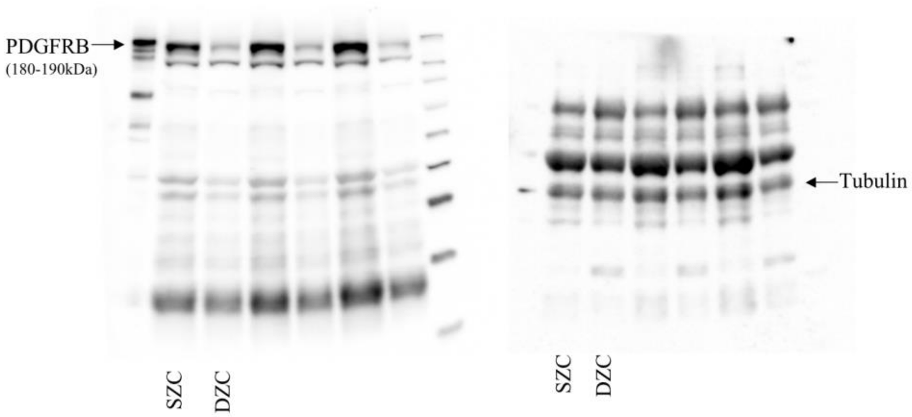

PDGFRB
(180-190kDa)
Tubulin
SZC
DZC
SZC
DZC
